# Book Review

**Published:** 1941

**Authors:** 


### Reviews of Books

**The Collected Papers of Wilfred Trotter, F.R.S.** Pp. vii., 194. London: Oxford University Press (Humphrey Milford). 1941. Price 10s. 6d.—In this small volume are collected together ten pre-war and two post-war essays, the former delivered by the late Mr. Trotter to various medical societies and subsequently published in various current medical journals. The appeal of these essays is by no means confined to the practitioners of the Art and Surgical Science of which Mr. Trotter was himself so distinguished a master. In his filial introduction Mr. W. R. Trotter says that his father here deals mainly with what may be called "the philosophy of medicine." He might with justice have gone farther and said that their appeal is to life itself, and therefore to all of us. In these essays Mr. Wilfred Trotter ranges from the emergencies of medical and surgical practice to the functions of the human skull; from the insulation of the nervous system to the possibility of intellect as a function, and from the art and science of a peaceful medicine to the "blood, tears and sweat" of bloody war. Over all, no matter how wide and varied their range, Mr. Trotter throws a spell of originality of thought, a wide knowledge of life, an equally broad intellectual outlook, and a human sympathy which combined, and in a word, are the real genius of Wilfred Trotter. It would be easy, did space not unhappily forbid, to quote from these papers many examples of the truth of these remarks. One alone must suffice, as topical as it is pregnant with thought. From "The Mind in War"—"the adversaries in the present war are separated not by mere political disagreement but by differences in their attitude towards life which practically amount to incompatible types of civilization . . . If the contest is of the kind suggested it is certain that the weaker must go to the wall." And is not this the law of an inexorable Nature? To read these essays in a collected form has been so great a privilege and pleasure that one can only hope that many others may have the same opportunity.

**Textbook on the Nursing and Diseases of Sick Children.** Third Edition. Edited by Alan Moncrieff, M.D., F.R.C.P. Pp. xiv., 639. Illustrated. London: H. K. Lewis & Co. Ltd. 1941. Price 21s.—Although primarily intended for nurses, this book contains much information of practical value to the medical student and even to the recently qualified practitioner. The editor admits that it exceeds the limits of the syllabus for the Sick Children's Nurses Certificate and this new edition, with its extra fifty pages, makes one wonder whether there may not be a danger of some contributors losing sight of its primary and essential aim, which is that of a text-book for nurses. There appears to be a certain lack of balance in some parts compared with others, an almost universal failing in books of multiple authorship. For instance, one might question the need for reference to a number of obscure diseases of bone in the orthopædic section, if no room can be found in the section on thoracic tuberculosis for such an important diagnostic procedure as gastric lavage. On the whole, however, serious omissions are quite exceptional, and it must be recognized that the book very adequately fills its position as the most useful and authoritative text-book of sick children's nursing now available. Among the many excellent illustrations a special welcome will be given to some more of Mr. Keogh's refreshingly realistic line drawings, which, together with the admirable new chapter on Children in the Tropics, must temper any regret one might have felt about the necessity for an increase in size.

**Leprosy.** Second Edition. By SIR L. ROGERS, K.C.S.I., C.I.E. M.D., F.R.C.P., F.R.C.S., F.R.S., I.M.S., and E. MUIR, C.I.E., M.D., F.R.C.P. Pp. xii., 260. Illustrated. Bristol: John Wright & Sons Ltd. 1940. Price 15s.—As tightly packed as a nodule with *Mycobacterium leprae* are these pages and eighty-one plates with most readable information on every aspect of a disease which affects between four and five million people. Africa, where incidence is as high as 130 per mille seems to have exported leprosy to Europe with Cambyses's soldiers, and to the New World with its slaves. In our present disillusionment it is gratifying to read that a high degree of civilization is inimical to the spread of leprosy. Nine years before Hansen's discovery in 1871 the Royal College of Physicians decided that leprosy was never communicable. But children and young folk up to twenty are the mainstay of the propagation of the disease by contact in the one-roomed hut method of living, especially where temperature and humidity are high and clothes few. Abrasions, scratches, insect bites and operation or postmortem pricks suffice as portals of entry. Fortunately the toxicity of the bacillus is low, for tissues may be crammed full yet symptoms slight or absent. The Cairo Congress (1938) confirmed the classification into Lepromatous (L 1, 2 and 3) and Neural (N 1, 2 and 3b) types and affirms that hydnocarpus oil and esters are still the most useful of the drugs which, however, only share pride of place in treatment with social and hygienic measures. Compulsion spells concealment, but consent means cure or care in agricultural, self-supporting settlements. Early and advanced cases should be separated and children segregated. Sterilization of married lepers and adoption of leper children is the commonsense method adopted in some asylums. Leprosy can be eradicated. Application lags behind the acquisition of scientific knowledge. This excellent book should renew the zeal of the leprologist and quicken the interest of the student.

**Venereal Diseases.** By E. T. BURKE, D.S.O., M.B., Ch.B. Pp. xv., 549. Illustrated. London: H. K. Lewis & Co. Ltd. 1940. Price 30s.—This book should hold a unique place amongst text-books on venereal diseases, because it represents the observations of one of the most brilliant venereologists of our time. The pictures, plates and photographs are excellent, but we should like to see more coloured plates of urethroscopic appearances in chronic urethritis. In some of his theories we find he is far ahead of the prevailing ideas on such subjects as the amount of treatment for early syphilis and the pathology of gonorrhœa. The author is most original in suggesting some of the causes for female gonorrhœa. Post-graduate students will find it a most excellent reference book, but it is too advanced for medical students, who might diminish their chances of a successful examination result by quoting some of Colonel Burke's theories.

**Technique of Gastric Operations.** By RODNEY MAINGOT, F.R.C.S. Pp. xii., 240. Illustrated. London: Oxford University Press (Humphrey Milford). 1941. Price 15s.—Modern gastric surgery has become so complicated, and so many methods are advocated by responsible surgeons, that there is room for this small, well illustrated, up-to-date manual. Clear details and advice are given as to the operative treatment of gastric diseases. We are glad to observe that the author stresses the importance of suction drainage if post-operative distension of the stomach or vomiting occurs. He also advocates suction-bronchoscopy to prevent post-operative pulmonary complications. He says that its use has almost abolished deaths from chest complications.

**A Complete Outline of Fractures.** By J. GRANT BONNIN, M.B., B.S., F.R.C.S., M.R.C.O.G. Pp. xii., 507. Illustrated. London: William Heinemann (Medical Books) Ltd. 1941. Price 25s.—A complete outline of fractures adequately describes this book, and it must approximate closely to the fulfilment of the aims of the author—that it should be the ideal students' text-book. Being an outline, it avoids most of the difficult practical problems which assail the practitioner. The generic term "exercises" is used to cover the key to treatment of all fractures around the shoulder joint, whereas the principle of relaxed circumduction exercises deserves a very full description for its practical application. The most recent methods are included with but few exceptions, the treatment of Colles' fractures in flexion being one of these. A somewhat unusual feature is the inclusion of quite a long chapter on fractures of the skull and the more important associated cerebral states. The chapters are well arranged, with a valuable opening section on the surgical anatomy of the part to be covered. The text is adequately and clearly illustrated, there is a short list of references at the end of each chapter, and the author's style makes easy reading. This book should be of great assistance to students, to whom it will furnish a very complete outline of fractures.

**Gynæcological Operations.** By J. LYLE CAMERON, M.D., F.R.C.S., F.A.C.S., M.R.C.O.G. Pp. xi., 200. Illustrated. London: Oxford University Press (Humphrey Milford). 1941. Price 21s.—The author of this book has endeavoured to describe the technique of gynæcological operations as clearly and concisely as possible, and he has been very successful in carrying out his task. The book covers the whole field of gynæcological operations, which have been arranged under five headings. (1) Pre-operative investigations; (2) vulval operations; (3) vaginal operations; (4) cervical operations; (5) abdominal gynæcological operations. In each section the indications and contraindications for any given operation are pointed out, together with the difficulties, dangers and complications which may occur. The operations themselves are very fully and carefully described and are accompanied with a number of very excellent drawings, illustrating the text. Under vaginal operations a very clear description of the Donald-Fothergill operation is given. Under cervical operations dilatation of the cervix and curetage are described, and it is pointed out that perforation of the uterns probably occurs more frequently than is generally recognized, and the importance of preventive treatment is stressed. Under the heading of abdominal gynæcological operations a full acount of the various methods of dealing with fibromyomata and malignant disease of the uterns is given, and the increasing tendency to treat malignant disease of the cervix with radium in preference to operation is pointed out. Among the complications of abdominal gynæcological operations it is stated that "burst abdomen" probably occurs in one per cent. of all abdominal operations, but if this is so, it must surely be due to some fault in the method of closing the abdominal wound. The book is attractively produced, well printed, indexed, and illustrated. It should be especially useful to those general surgeons who are interested in gynæcological operations.

**Fractures and other Bone and Joint Injuries.** Second Edition. By R. WATSON-JONES, M.Ch., F.R.C.S. Pp. xii., 724. Illustrated. Edinburgh: E. & S. Livingstone. 1941. Price 50s.—This excellent book on fractures came out in its second edition one year from it's original publication, and a reprint now follows within a further six months. These facts vouch for its deservedly popular reception. In the second edition Mr. Watson-Jones has re-written the chapter on compound fractures and war wounds, and includes recent developments in chemo-therapy, and blood and plasma transfusion in the treatment of shock and hæmorrhage. With his usual clarity he has covered this whole subject exceedingly well. There are other minor improvements to be found, and the reputation of this book cannot but be enhanced by the appearance of this new edition. The illustrations remain an outstanding feature and will be found to be of tremendous assistance when studying the text.

**The Early Treatment of War Wounds.** By WILLIAM ANDERSON, O.B.E., F.R.C.S. Pp. xi., 96. Illustrated. London: Oxford University Press (Humphrey Milford). 1941. Price 5s.—This small book is one of the Oxford War Primers, under the general editorship of Lord Horder. In one sense this series represents a revision of that published during the last war and is written by men who had experience of that catastrophe. The opening chapters deal very largely with matters of organization, of advanced dressing stations and casualty clearing stations. It is not quite clear to what extent the distinction of these units will be applicable to affairs in the present war. There is then a brief description of the principles of treatment of wounds, wounds of the brain, penetrating wounds of the abdomen and penetrating wounds of the thorax. The last chapters deal with wounds of the joints, vertebral column, nerves and arteries. The possibilities of combating wound infection by drugs of the sulphonamide group are hinted at, rather briefly, as are the modern methods of blood transfusion. The small volumn contains a great deal of useful information, packed into a small space.

